# Retraction: Combination of Tanshinone IIA and Cisplatin Inhibits Esophageal Squamous Cell Carcinoma by Down-Regulating NF-kB/COX-2/VEGF Pathway

**DOI:** 10.3389/fonc.2021.721658

**Published:** 2021-06-29

**Authors:** Frontiers Editorial Office

**Affiliations:** Frontiers Media SA, Lausanne, Switzerland

The Journal retracts the September 10, 2020 article cited above for the following reason:

Following publication, concerns were raised regarding the integrity of the images in the published figures. The authors failed to provide a satisfactory explanation during the investigation, which was conducted in accordance with Frontiers’ policies.

This retraction was approved by the Chief Editors of Frontiers in Oncology and the Chief Executive Editor of Frontiers. The authors did not agree to this retraction.

